# New Strategy Based on Click Reaction for Preparation of 3-Acyl-4-hydroxycoumarin-Modified Silica as a Perspective Material for the Separation of Rare Earth Elements

**DOI:** 10.3390/molecules31020369

**Published:** 2026-01-20

**Authors:** Dzhamilya N. Konshina, Ekaterina S. Spesivaya, Ida A. Lupanova, Anton S. Mazur, Valery V. Konshin

**Affiliations:** 1 Department of Chemistry and High Technologies, Kuban State University, 149 Stavropolskaya Str., 350040 Krasnodar, Russia; jfox@list.ru (D.N.K.); ekaterina-1305@mail.ru (E.S.S.); konovalova_ida@mail.ru (I.A.L.); 2 Center for Magnetic Resonance, Saint Petersburg State University, 198504 St. Petersburg, Russia; a.mazur@spbu.ru

**Keywords:** silica, rare earth elements, 3-acyl-4-hydroxycoumarin, preconcentration

## Abstract

The separation of rare earth elements (REEs) with similar chemical properties remains a relevant challenge today, most often addressed using liquid–liquid and solid-phase extraction with various chelating agents. Excellent complexing agents for REEs are 1,3-diketones and their analogs. We have for the first time proposed a method for preparing a material consisting of a covalently immobilized 3-acyl-4-hydroxycoumarin ligand on silica. For its synthesis, we employed a strategy based on the “click” reaction of 3-azidopropyl silica with a propargyl-containing coumarin–chalcone conjugate—this approach is the most tolerant and does not affect the coordinationally active fragment of the ligand. The material was characterized by thermal analysis, IR spectroscopy, and ^13^C NMR. The potential of the synthesized material for REE preconcentration was demonstrated at pH 5–5.5: high extraction efficiency for Gd(III), Dy(III), Er(III), Eu(III), Sm(III), and Yb(III) was observed, with fast adsorption kinetics (30 min) and extraction degrees of ~98%. Under unified conditions of static and dynamic extraction for Gd(III), Dy(III), Er(III), Eu(III), Sm(III), and Yb(III), affinity series toward the surface were obtained as a function of the distribution coefficient. It was shown that 10-fold molar excesses of Fe(III), Al(III), Cu(II), Ni(II), and Co(II) allow retention of more than 95% extraction for Dy(III) and Er(III). After adsorption of Dy(III) and Er(III), shifts in the carbonyl group absorption bands are visible in the IR spectra of the material, indicating a chelating mechanism of sorption. Additional studies are required for implementation in analytical and preparative REE separation schemes; however, preliminary data show that the material is a highly active adsorbent.

## 1. Introduction

**Figure 1:**
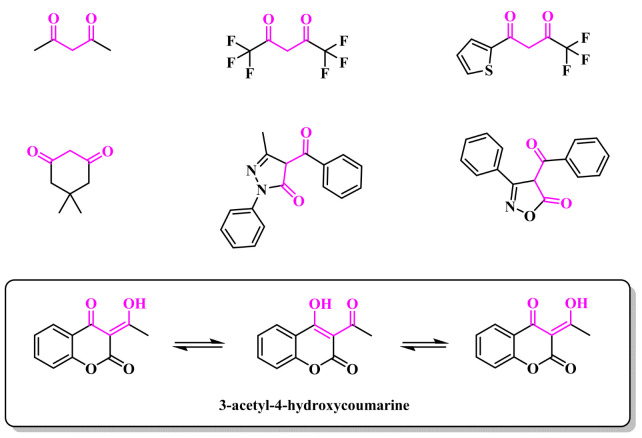
Structures of various β-diketone ligands.

Rare earth elements are the cornerstone of many modern technologies; without their unique properties, it is difficult to imagine such fields as electronics, energy, medical diagnostics, and others [1]. Over the past 50 years, virtually the entire spectrum of some of the “youngest” non-radioactive elements has transformed from exotic representatives of the periodic table into a sought-after commodity. The isolation of rare earth elements from natural and secondary raw materials, as well as their separation, is one of the interesting problems of the present time, with a huge amount of research devoted to this topic [2,3,4,5,6]. Significant advances have been achieved using liquid–liquid extraction [7,8,9] and various sorption methods [10,11,12,13,14,15]. Both approaches aim to develop effective ligands or materials containing covalently immobilized chelating fragments. Among the most promising ligand types are various compounds containing a 1,3-dioxo motif in their structure: classical aliphatic 1,3-diketones [16,17], their fluorinated analogs [17,18,19,20], and heterocyclic compounds in which an oxo group is linked to the ring, such as 4-acylpyrazolones [21,22,23] and 4-acylisoxazolones [24,25] (Figure 1). Methods for synthesizing such compounds are well-developed [26,27], and their coordination properties have been studied in detail [28]. Among this diversity of ligands, the least studied are the structurally related 3-acyl-4-hydroxycoumarins, which exhibit rich tautomerism [29] that determines their strong coordination ability [30], including toward rare earth elements [31,32,33].

**Figure 2:**
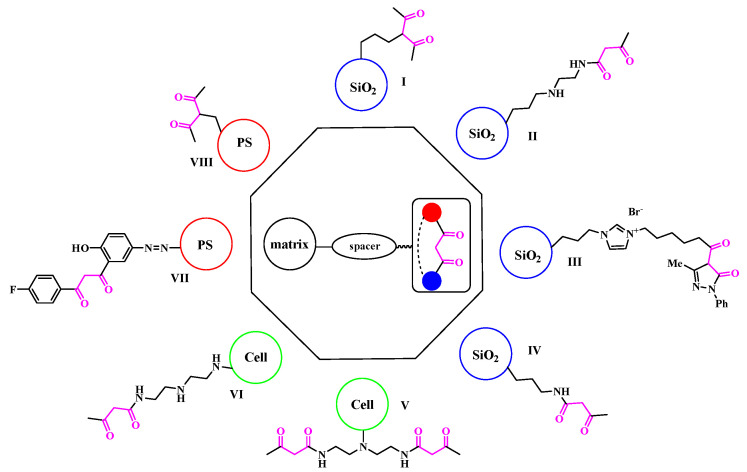
Materials with grafted 1,3-dioxo fragment.

Sorption materials containing a covalently immobilized structural motif of 1,3-dicarbonyl compounds are known in quantities several orders of magnitude smaller than the corresponding molecular ligands. Materials based on silica (**I**–**IV**), cellulose (**V**, **VI**), and polystyrene (**VII**, **VIII**) are described (Figure 2). Alkylation [34,35,36,37], azo coupling [38], and acylation with diketene [39,40,41] reactions are used for their synthesis. The review [42] examines the preparation of materials with 1,3-diketone fragments using sol–gel technology.

It is well-known that the covalent immobilization of a ligand on the surface of a solid matrix rigidifies the chemical structure of the complexing agent, thereby restricting its steric and conformational flexibility and its ability to adapt to the electronic structure of the metal ion. This results in altered selectivity for the extraction of rare earth ions with similar properties. Immobilization of ligands on silica surfaces is accompanied by the formation of strong siloxane bonds between the linker and the matrix, which imparts significantly higher chemical stability to the resulting materials and enhances their potential for regeneration and reuse. The objective of this work is to develop a procedure for covalently attaching the 3-acyl-4-hydroxycoumarin moiety to the silica surface, verify the success of the immobilization, and assess the sorption properties of the synthesized material to Gd(III), Dy(III), Er(III), Eu(III), Sm(III), and Yb(III).

## 2. Results and Discussion

### 2.1. Synthesis of Material

**Figure 3:**
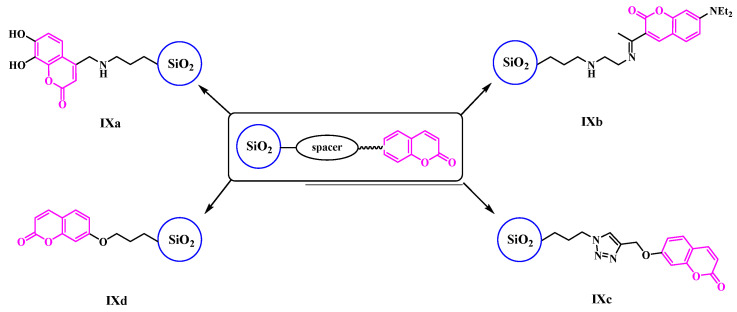
Materials with grafted coumarin skeleton.

By the beginning of our research, a number of materials containing a covalently immobilized coumarin skeleton IXa [43], IXb [44], IXc [45,46,47,48,49,50], IXd [51] had been described (Figure 3).

However, materials based on immobilized 3-acyl-4-hydroxycoumarin ligands were unknown.

The rational design of the target material involves positioning the anchoring group in the ligand molecule as far as possible from the coordinating moiety to minimize steric hindrance during complexation. The presence of reactive hydroxyl and oxo groups in 3-acyl-4-hydroxycoumarin restricts the range of selective reactions available for covalent attachment, such as alkylation reactions (as in the cases of **IXa** and **IXd**) or condensation reactions (as in the case of I**Xb**). We considered that a click-assembly strategy for synthesizing the target material via a [3 + 2] azide–alkyne cycloaddition reaction would be optimal [52,53,54].

**Scheme 1:**
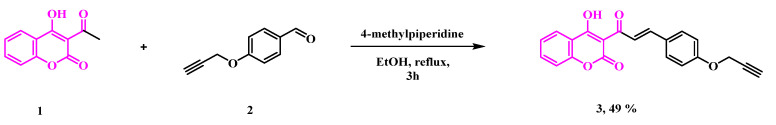
Synthesis of clickable coumarin chalcone.

When selecting a strategy for synthesizing a clickable acylcoumarin derivative, we primarily considered the availability of starting materials, the ease of the reactions, and their reproducibility. Accordingly, we chose to employ a coumarin chalcone derivative 3 [55], which can be readily synthesized in two steps from commercially available 4-hydroxycoumarin using a known acylation protocol [56], followed by coupling with 4-(prop-2-yn-1-yloxy)benzaldehyde [55,57,58] (Scheme 1).

**Scheme 2:**
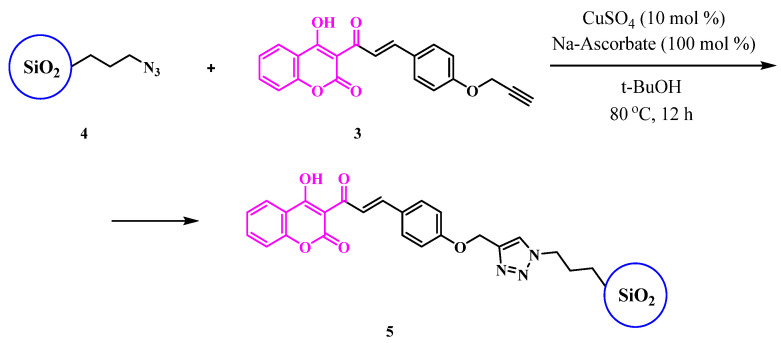
Grafting of coumarin chalcone.

The grafting of coumarin chalcone **3** by reaction with 3-azidopropyl silica **4** [59] was carried out in *tert*-butanol at 80 °C for 12 h in the presence of 10 mol.% copper (II) sulfate and 100 mol.% sodium ascorbate (Scheme 2).

### 2.2. Characterization of Material

**Figure 4:**
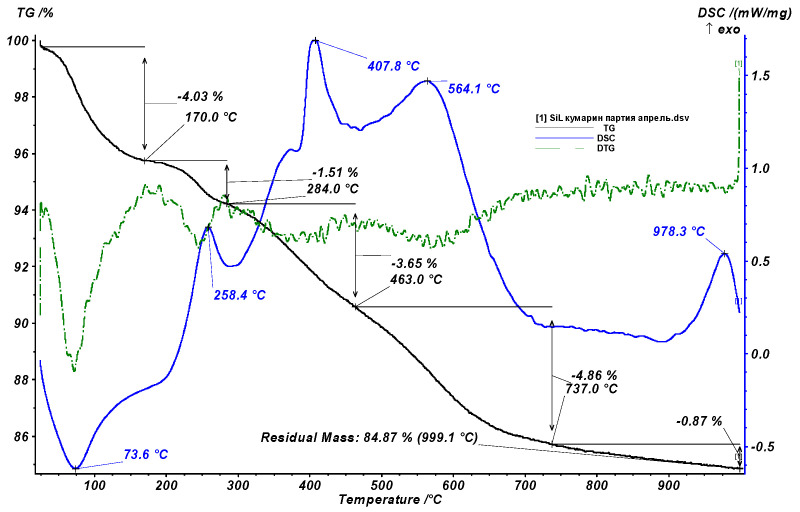
Thermogravimetric curves for the modified silica **5**.

**Figure 5:**
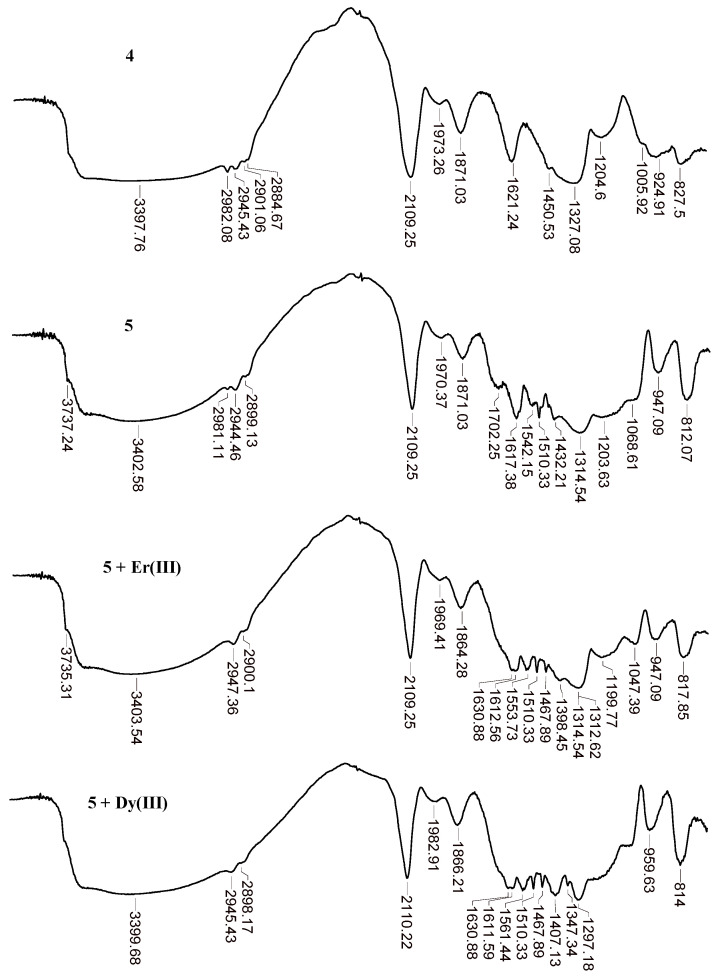
Evolution of IR spectra during the synthesis of silica **5** and its reaction with Er(III) and Dy(III).

**Figure 6:**
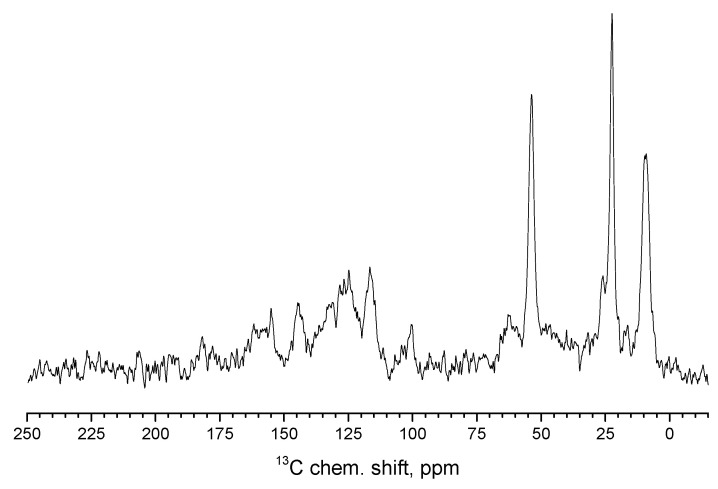
^13^C NMR spectrum for the organomineral material **5**.

The synthesized organomineral material **5** was characterized by thermogravimetric analysis (TGA) (Figure 4), IR spectroscopy (Figure 5), and solid-state ^13^C NMR spectroscopy (Figure 6). The thermogram reveals four main stages of mass loss. Upon heating from 20 to 170 °C, physically adsorbed water and residual solvent are removed, resulting in an endothermic effect observed in the DSC curve and a relative mass loss of 4.03%. The subsequent stages of mass loss in the temperature range of 200–750 °C are accompanied by exothermic peaks in the DSC curve, indicating thermal degradation of the grafted organic layer. Over the entire range from 130 to 1000 °C, a total mass loss of 10.8% is observed, attributable to the destruction and combustion of the organic functional layer. The exothermic peak in the DSC curve at 978 °C corresponds to the formation of the crystalline SiO_2_ phase (cristobalite).

The diffuse reflectance IR spectrum of the modified silica **5** exhibits a broad absorption band in the range of 3000–3700 cm^−1^, attributable to the superposition of stretching vibrations of Si–OH groups and H–O–H vibrations of adsorbed water molecules involved in both intra- and intermolecular hydrogen bonding. A strong band at 1970 cm^−1^ corresponds to overtones of the fundamental Si–O–Si vibrations. Characteristic bands at 1702 cm^−1^ arise from the stretching vibrations of the C=O group, while those at 1617 cm^−1^ are assigned to the stretching vibrations of the C=N group. Additionally, intense absorption bands at 1545 and 1510 cm^−1^ correspond to the stretching vibrations of C(sp^2^)–C(sp^2^) bonds in the aromatic rings of the grafted moieties

As seen from the IR spectroscopy data (ν N_3_ = 2109 cm^−1^), complete conversion of the azide groups does not occur. This can likely be attributed to the high steric bulk of the coumarin–chalcone ligand, which hinders the quantitative progress of the reaction.

In the diffuse reflectance IR spectra of the silica sample after reaction with an Er(III) and Dy(III), a shift in the absorption band corresponding to the C=O group to the low-frequency region at 1630 cm^−1^ is observed, which is associated with the delocalization of the electron density due to its participation in the coordination of the Er(III) and Dy(III) ions.

Comparing the IR spectra of silica 5 before and after sorption of Ln(III), a decrease in the ν (C=O) values can be observed, which confirms extraction via a coordination mechanism accompanied by the formation of a covalent C–O–Ln(III) bond (Supplementary Materials, Figures S1–S4).

The ^13^C NMR spectrum (Figure 6) of the synthesized organomineral material **5** contains two groups of signals are observed: those of the aliphatic carbon atoms of the propyl fragment of the linker and the methylene group in the 5–60 ppm range, as well as signals of the Csp^2^ aromatic ring, vinyl, and carbonyl groups in the 100–170 ppm range.

### 2.3. Adsorption Properties of Material

#### 2.3.1. Effect of pH

**Figure 7:**
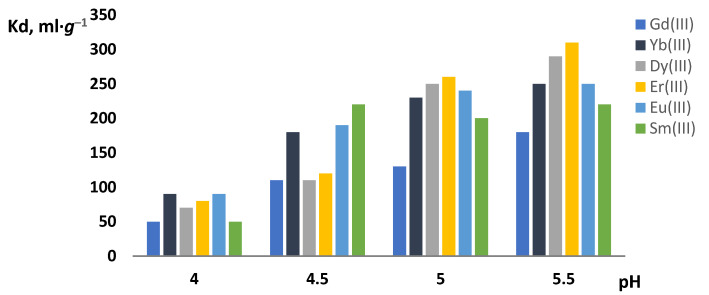
Distribution coefficients of Gd(III), Dy(III), Er(III) (Eu(III), Sm(III), Yb(III) (C = 3.5 × 10^−4^ mol·L^–1^) as a function of pH (m (silica) = 50 mg, t = 2 h).

The tendency of lanthanide ions to undergo hydrolysis increases with increasing atomic number and decreasing ionic radius. For example, at pH 6, the proportion of free aquated cations is approximately 75% for Ce^3+^ and Pr^3+^, 60% for Nd^3+^ and Eu^3+^, and only 50% for Sm^3+^ [60]. Accordingly, pH 5.5 was selected as the upper limit for the aqueous phase. The extraction of Sm(III), Eu(III), Dy(III), Er(III), Yb(III), and Gd(III) ions, expressed in terms of the distribution coefficient, increased with increasing pH in the range of 3.5–5.5, reaching a maximum at pH 5.5 (maintained using an acetate buffer solution); the solid–liquid contact time was 2 h (Figure 7). For surface-modified silica, it should be noted that the grafted ligands may not form an ideal monolayer but rather create “islands” on the surface. Furthermore, residual silanol groups located between the ligand moieties can mediate Ln^n+^–HO–Si interactions through Van der Waals forces, electrostatic interactions, or coordination bonds, which may play a significant role in solid-phase extraction. The contribution of nonspecific sorption on the unmodified silica surface under the selected conditions did not exceed 10%, as confirmed experimentally. To accurately determine this value, the initial Ln(III) concentration was kept at the same level as in the experiments with the modified silica.

The tendency of lanthanide ions to undergo hydrolysis increases with increasing atomic radius and decreasing ionic radius. For example, at pH 6, the proportion of free cations is 75% for Ce^3+^ and Pr^3+^, 60% for Nd^3+^ and Eu^3+^, and only 50% for Sm^3+^ [60]. Therefore, 5.5 was chosen as the upper limit of the aqueous phase pH. The extraction of Sm(III), Eu(III), Dy(III), Er(III), Yb(III), and Gd(III), as a function of the partition coefficient, increased with changing solution pH in the range 3.5–5.5, reaching a maximum at 5.5, maintained with an acetate buffer solution; the phase contact time was 2 h (Figure 7). In the case of surface-modified silica, it is necessary to consider that the grafted ligands may not form an ideal monolayer on the surface, but rather create “islands.” Moreover, residual silanol groups are located between the ligand fragments, mediating Me^n+^–HO-Si interactions via van der Waals, ionic, or coordination interactions, which may play an important role in the solid-phase extraction. The contribution of nonspecific sorption by the surface of unmodified silica under the chosen conditions did not exceed 10%, which we confirmed experimentally. To accurately estimate this relative value, the initial Ln(III) concentration was maintained at the same level as in the experiments with modified silica.

#### 2.3.2. Effect of Contact Time on Metal Adsorption

**Figure 8:**
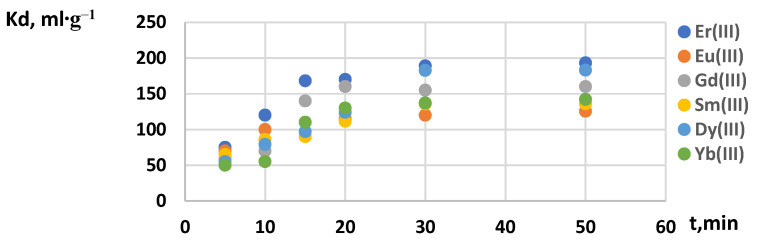
Distribution coefficients of Gd(III), Dy(III), Er(III) (Eu(III), Sm(III), Yb(III) (C = 3.5 × 10^–4^ mol·L^–1^) as a function of the phase contact time (m (silica) = 50 mg, pH 5.5).

To investigate the effect of phase contact time on the adsorption process, experiments were conducted with contact times varying from 5 to 50 min. The dependence of the Ln(III) distribution coefficient and adsorption capacity on contact time was plotted (Figure 8). Equilibrium time is a relatively straightforward parameter to determine. Since the sorption process involves a heterogeneous reaction between the grafted functional groups and Ln(III) ions, its rate (and thus the required time) depends on the initial concentrations, the liquid-to-solid ratio, and the molar ratio of the interacting components. Accordingly, the initial Ln(III) concentration was selected to achieve an approximately 1:1 molar ratio between the functional groups on the sorbent and the Ln(III) ions in the solution. The number of functional groups was calculated from thermogravimetric analysis data, assuming that the total mass loss in the temperature range of 130–700 °C corresponds to the combustion of the organic functional layer. As shown in Figure 8, both the distribution coefficient and the adsorption capacity increase with contact time, reaching a plateau after 30 min. This indicates that equilibrium has been attained in the sorbent–sorbate system and that the available binding sites on the silica surface are fully occupied.

#### 2.3.3. Adsorption Isotherms

**Figure 9:**
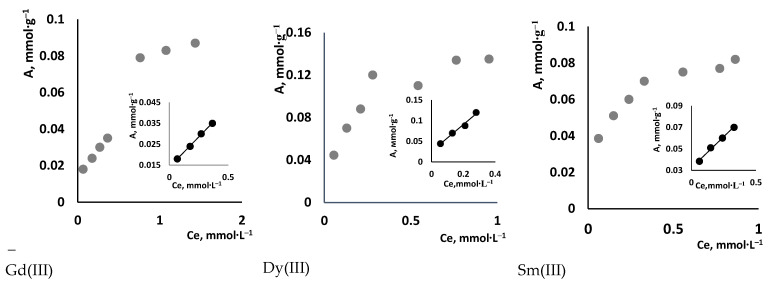
Sorption capacity of the silica **5** as a function the Gd(III), Dy(III), Sm(III) concentration in solution.

When describing the sorption capacity of silica modified with grafted complexing groups, it is important to recognize that the interaction of metal ions with chelating sorbents is highly complex. Local concentrations of reactants in the near-surface layer of the sorbent are abnormally high, their distribution across the surface and within the material is nonuniform, and key phenomena include lateral interactions between grafted ligands, distortion of the coordination sphere of immobilized metal complexes, the influence of the surface electrostatic potential, the chemical nature and concentration of electrolytes in the liquid phase on surface reactions, and the formation of multiple types of surface complexes rather than a single one. In complexation with grafted ligands, the most stable complexes are those whose stoichiometry provides the optimal balance between the chelate effect and steric hindrance arising from ligand immobilization. The influence of the initial concentrations of Sm(III), Eu(III), Dy(III), Er(III), Yb(III), and Gd(III) on the sorption activity of the grafted chelating groups was described by type I sorption isotherms (convex upward, according to the Giles classification) [61] (T = 298 K) (Figure 9). The isotherms were constructed by plotting sorption capacity against the equilibrium concentration of Ln(III) in solution, with the Henry region (linear portion at low concentrations) separately identified for calculation of the distribution coefficient Kd (Table 1). The maximum sorption capacity Amax, representing the static sorption capacity of the sorbent toward the extracted metal ion, was determined from the plateau (horizontal section) of the isotherm.

**Figure 10:**
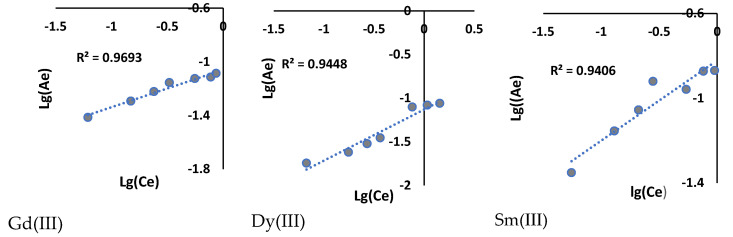
Equilibrium isotherm data (Freundlich isotherm model) for the adsorption of Gd(III), Dy(III), Sm(III) on modified silica.

Considering the heterogeneity of the silica surface resulting from the “island-like” grafting of functional groups and the incomplete nature of the immobilization reactions, the Freundlich model—which is commonly employed to describe the sorption of metal ions on various ion-exchange materials and chelating sorbents—was found to provide the best fit for the adsorption equilibrium data [61]. Linearized plots (anamorphoses) derived from the integral equilibrium isotherms in the coordinates of the Freundlich equation exhibited high coefficients of determination (R^2^ > 0.99) (Figure 10). In contrast, when the sorption isotherms were processed using the coordinates of the Langmuir and Temkin models, the coefficients of determination did not exceed 0.87.

The ligand affinities for rare earth element (REE) ions and the chemical properties of the lanthanoids themselves are very similar due to the close values of their ionic radii (for example, Sm^3+^ 0.96 Å, Eu^3+^ 0.95 Å, Gd^3+^ 0.94 Å, Dy^3+^ 0.91 Å, Er^3+^ 0.89 Å, Yb^3+^ 0.89 Å [62]). Ln(III) ions are considered strong Lewis acids; they readily coordinate, in both neutral and acidic media, with hard electron donors such as oxygen-containing (O,O-) ligands [63].

One approach that allows differentiation of the coordination—and correspondingly the extraction—behavior of Ln(III) ions is the covalent immobilization of an effective O,O-ligand on a solid surface. This makes the chemical structure of the complexing agent more rigid, thereby restricting its steric and geometric freedom and its ability to adapt to the electronic structure of the ion, ultimately altering the selectivity for extracting ions with similar properties.

**Figure 11:**
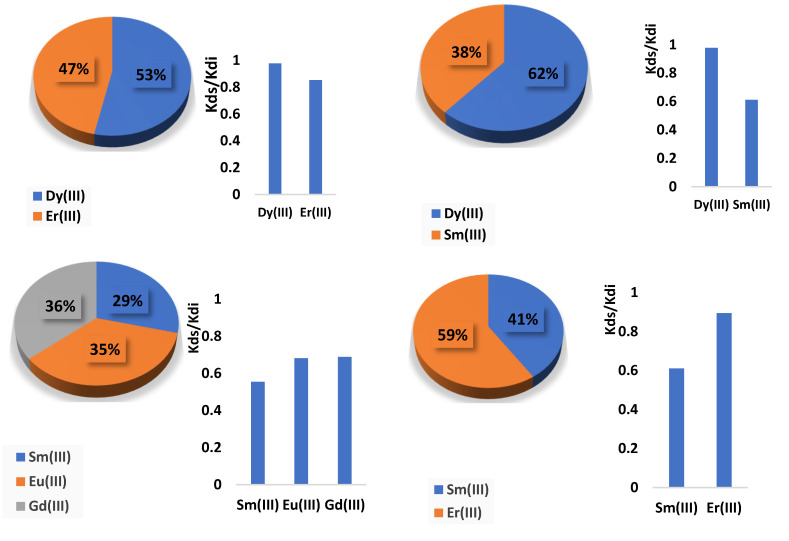

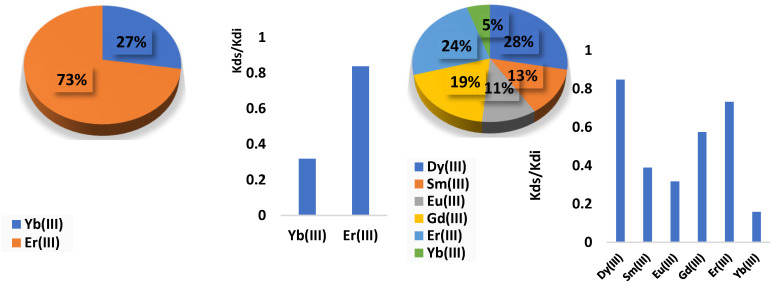
Ratio of distribution coefficients and sorption capacity of Gd(III), Dy(III), Er(III) (Eu(III), Sm(III), Yb(III) (C = 3 × 10^−4^ mol/L) in multicomponent system relative to individual solutions (Kds/Kdi), (As/Amax, %).

The calculated distribution coefficients (Table 1) demonstrate the selectivity of the modified surface toward the selected rare earth ions, which can be expressed as a surface affinity series. The distribution coefficient values increase with increasing ionic radius of the rare earth ions (i.e., with increasing basicity of the aquated cations), although deviations are observed for Gd(III) and Yb(III). For subsequent modeling of binary, ternary, quaternary, and senary systems and for assessing mutual competitive effects, the initial Ln(III) concentrations were selected from the Henry region to enable reliable comparison of changes in surface affinity during the transition from single-component to multicomponent systems. We have previously described this algorithm for studying competitive sorption [64]. Based on these data, the following systems were modeled: Er(III)–Dy(III), Dy(III)–Sm(III), Sm(III)–Eu(III)–Gd(III), Er(III)–Yb(III), Er(III)–Sm(III), Dy(III)–Yb(III)–Er(III), Sm(III)–Eu(III)–Dy(III), and Dy(III)–Yb(III)–Er(III)–Sm(III)–Eu(III)–Gd(III). For the Er(III)–Dy(III) pair, where the individual distribution coefficients are similar, no significant competition or decrease in extraction efficiency is observed, as evidenced by the comparable Kd values obtained in the multicomponent system and in the individual adsorption equilibria within the Henry region. The calculated distribution coefficients are presented as diagrams that allow evaluation of changes in this parameter in multicomponent systems relative to single-component solutions (Kds/Kdi) and assessment of the contribution to sorption capacity under competitive conditions (Figure 11).

For the Dy(III) –Sm(III), suppression of Sm(III) extraction and a decrease in the Kd value are observed compared to individual extraction. In the Sm(III)–Eu(III)–Gd(III) system, a more than 1.5-fold decrease in Kd for Eu(III) and Gd(III) is observed. In the case of an equimolar six-component mixture, a more than ten-fold decrease in Kd for Yb(III) is observed.

The values of maximum capacities and distribution coefficients obtained on model solutions under static and dynamic conditions are comparable to those achieved even on mesoporous silica grafted with O,O-ligands (Kd = 950 mL·g^−1^) and extraction capacity (1 mg·g^−1^) [65].

#### 2.3.4. Dynamic Preconsentration

Dynamic sorption enables the separation and preconcentration of substances in a flow-through mode, which is of particular interest because it eliminates the need for phase separation after preconcentration and facilitates process automation. The results of dynamic sorption experiments with the modified silica are presented as breakthrough curves, which show the dependence of the relative sorbate concentration (C/C_0_) in the effluent on the volume of solution passed through the column at a constant flow rate. The distribution coefficients (Kd) of Ln(III) ions were calculated for Gd(III), Dy(III), Er(III), Eu(III), Sm(III), and Yb(III) from individual solutions (C_0_ = 3 × 10^−6^ M for each ion, V = 50 mL, solution flow rate = 1.25 mL/min). The calculated Kd values (Table 2) allow construction of an affinity series for the ions toward the modified surface, which is not reversed and exhibits a similar trend to that observed in static mode: Er(III) > Dy(III) > Sm(III) ≈ Eu(III) ≈ Gd(III) > Yb(III).

**Figure 12:**
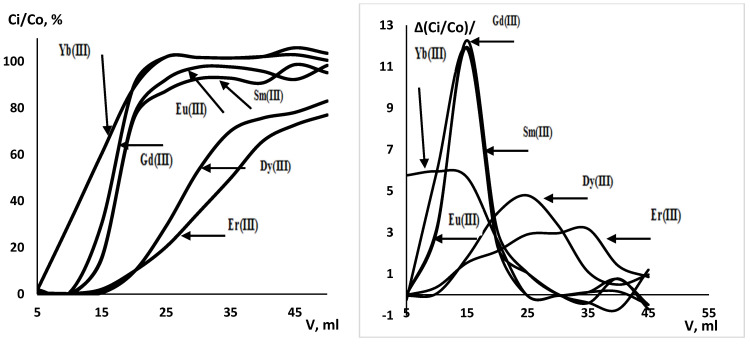
Dynamic breakthrough curve of Gd(III), Dy(III), Er(III) (Eu(III), Sm(III), Yb(III) (pH = 5.5, V = 1.25 mL/min, and m(sorbent) = 50 mg).

Taking into account the changes in the distribution coefficient for individual systems, a six-component system containing Gd(III), Dy(III), Er(III), Eu(III), Sm(III), and Yb(III) in equimolar amounts was simulated and frontal dynamic breakthrough curve were constructed (Figure 12). The calculated distribution coefficient (Table 3) indicates the mutual competitive influence of Ln(III) and the suppression of the extraction of Yb(III), Gd(III), Eu(III), and Sm(III), but the possibility of separation by REE ion groups Er(III) + Dy(III) > ~Eu(III) ~ Gd(III) Sm(III) Yb(III) remains.

At 10-fold molar excesses of Fe(III), Al(III), Cu(II), Ni(II), Co(II) in a six-component system, an even more significant suppression of the sorption of Yb(III), Sm(III), Gd(III), Eu(III) can be observed than in the case of a six-component system without interfering cations. It is interesting to note that in the case of sorption from six-component systems under conditions of the addition of 10-fold molar excesses of Fe(III), Al(III), Cu(II), Ni(II), Co(II) in a static mode, an even greater decrease in the values of the distribution coefficients of Yb(III), Sm(III), Eu(III) can be observed.

## 3. Materials and Methods

MN Kiesegel 60 silica (Macherey-Nagel)(GmbH and Co.KG, Duren, Germany) (40–60 μm fraction, Ssp = 327.8 m^2^/g (after removing traces of water) and total pore volume (Vpore = 0.095 cm^2^/g, dominant pore diameter 1.77 nm) was used as the matrix for functionalization. Standard solutions of Ln(III) [Gd(III), Dy(III), Er(III), Eu(III), Yb(III), Sm(III)] with a concentration of 5·10^−2^ M were prepared by dissolving accurately weighed portions of the corresponding oxides in 10% HCl. Working solutions were prepared by dissolving standard solutions. The solution-to-solid ratio was set at 1000 (V/m). Samples (50 mg) were mixed on an orbital shaker, then the supernatant was collected and analyzed. All experiments were performed in triplicate; only average values are presented. IR spectra were recorded on a Shimadzu Prestige-21 IR spectrometer (Shimadzu, Tokyo, Japan) in the wavelength range of 4000–400 cm^−1^ with a DRS-8000 diffuse reflectance attachment.

Solid-state ^13^C NMR spectra of the modified silica **5** were recorded at the Magnetic Resonance Center of St. Petersburg State University on a Bruker Avance III 400 WB spectrometer (frequency 100.64 MHz for ^13^C) (Bruker, Karlsruhe, Germany) at room temperature using a Bruker CP/MAS probe with a 4 mm rotor diameter. Spectra were recorded at a rotor speed of 10 kHz using the CP TOSS (polarization transfer with rotational sideband suppression) sequence. Thermal analysis of the silica sample was performed on an STA 409 Luxx instrument (Netzsch) (Netzsch-Gerätebau GmbH, Selb, Germany) in platinum–rhodium microcrucibles over a temperature range of 30–1000 °C in air (50 mL/min) at a heating rate of 10 °C/min.

The pH of all working buffer solutions was monitored with an Expert-001 ion meter (Ekoniks-ekspert, Moscow, Russia) using a calibrated ESC-10608 combined glass (Ekoniks-ekspert, Moscow, Russia) electrode.

The residual concentration of individual metals in solution after sorption experiments was monitored photometrically with an SS2107UV spectrophotometer (LEKI Instruments, Finland) at a 10 mm optical path length, using a spectrophotometric method based on the reaction of rare earth elements with arsenazo(III) [63]. The residual concentration of metals in the combined state in the solution after the sorption experiments was monitored using an iCAP 6000 (Thermo Fisher Scientific, Waltham, MA, USA) inductively coupled plasma optic emission spectrometer. The analytical lines of the elements used for the determination were as follows: Gd(III)—335.047 nm; Dy(III)—353.170 nm; Er(III)—323.058 nm; Eu(III)—381.963 nm; Sm(III)—330.639 nm; Yb(III)—328.937 nm.

### 3.1. Synthesis 3-Acyl-4-hydroxycoumarin-Modified Silica **5**

Prepare 0.1 g (0.288 mmol) of hydroxy-3-(3-(4-(prop-2-yn-1-yloxy)phenyl)acryloyl)-2H-chromen-2-one in a thick-walled test tube equipped with a magnetic stirrer and a fluoroplastic screw cap. Add 12 mL of tert-butanol and 2 g of 3-azidopropyl-silica (azido group capacity 0.5 mmol/g). Then, with stirring, add 288 μL of 0.1 M CuSO_4_ solution (0.0288 mmol, 10 mol%) and a solution of 57 mg (0.288 mmol, 100 mol%) sodium ascorbate in 500 μL of distilled water. The reaction mixture was maintained at 80 °C with vigorous stirring for 12 h. Then the silica was filtered, washed with hot ethanol, acetone, and Soxhlet ethanol extraction, and dried in vacuum at a residual pressure of 1 mmHg to constant weight. A canary-yellow powder was synthesized.

### 3.2. Adsorption Experiments

The distribution coefficient in static conditions (Kd), distribution factor (SF), and recovery (R) were calculated.

(1) $$K_{d}=\frac{{(C}_{ai\;}-C_{ae\;})\times V}{C_{ae}\times m}$$

(2) $$SF=\frac{K_{dM1}}{K_{dM2}}$$

(3) $$R=\frac{C_{se}}{C_{ai}}$$

The following equations were adhered to:

where

C_ae_—equilibrium concentration of metal ions in the aqueous phase;

C_ai_—initial concentration of metal ions in the aqueous phase;

C_se_—equilibrium concentration in the sorbent phase.

V—volume of the solution, L; m—the mass of the sorbent, g.

The distribution coefficient under dynamic conditions was calculated (4):

(4) $$Kd=w\times \frac{t_{0.5Co}-t_{k}}{m}$$

w—volumetric flow rate of the solution, cm^3^/min;

t_0.5 Co_—time corresponding to the half-concentration yield of the extracted REE ion, min;

t_k_—time it takes to fill the column’s free volume, min;

m—mass of the sorbent, g.

The effect of the pH: A solution containing 20 μg of Ln(III) and 50 mg of sorbent was added to 50 mL of a solution with a given pH value and shaken for 120 min. The sorbent was then filtered, and the residual concentration of Ln(III) in the solution was determined using the Arsenazo III photometric method.

A sorption isotherm: A series of solutions with a pH of 5.5 were prepared. A total of 50 mL of acetate buffer was added to a 100 mL flask; an aliquot of Ln(III) solution with an initial concentration of 1 × 10^−4^–1.2 × 10^−3^ M and 50 mg of sorbent was added, and the mixture was stirred for 24 h. Ln(III) was determined photometrically with Arsenazo III [66].

The sorption capacity of the materials was calculated (5) as follows:

(5) $$A=\frac{{(C}_{ai}-C_{ae})\times V}{C_{ae}}$$

where

C_ae_—the equilibrium concentration of metal ions in the aqueous phase;

C_ai_—initial concentration of metal ions in the aqueous phase, mmol/L;

V—volume of the solution, L;

m—mass of the sorbent, g.

Distribution coefficients (Kd) and separation factors (SF) of Sm(III), Eu(III), Dy(III), Er(III), Yb(III), and Gd(III): A series of solutions with pH 5.5 was prepared. A total of 50 mL of acetate buffer was added to a 100 mL l flask; an initial concentration of Ln(III) of 1 × 10^−4^ to 5 × 10^−4^ M, and 50 mg of the sorbent was added; it was stirred for 24 h. The Ln(III) content in the supernatant after equilibrium was established and was determined by OES-MS.

Column Study: The column (diameter = 0.5 cm) bottom was filled with glass wool to support the adsorbent, then 0.2 g of modified silica was placed in the column, with the layer height being 0.7 cm. Solutions of Sm(III), Eu(III), Dy(III), Er(III), Yb(III), and Gd(III) were passed through the column at a constant flow rate of 1.25 mL/min. Aliquots were collected in 5 mL, and Ln(III) was determined by OES-MS.

## 4. Conclusions

In this study, we describe the synthesis and comprehensive characterization of a novel organosilica material, featuring covalently grafted 3-acyl-4-hydroxycoumarin moieties on the silica surface. At pH 5.0–5.5, the material exhibits high extraction efficiency toward Gd(III), Dy(III), Er(III), Eu(III), Sm(III), and Yb(III) ions, with rapid adsorption kinetics (equilibrium reached within 30 min). Due to steric hindrance of the immobilized ligand, complete conversion of surface azide groups does not occur, resulting in the formation of “islands” containing both 3-acyl-4-hydroxycoumarin and residual N_3_ fragments. In contrast to residual silanol groups, which can ionize and coordinate metal ions, these islands show negligible nonspecific sorption toward the studied lanthanide ions. The heterogeneity of the grafted surface is indirectly confirmed by sorption isotherm analysis: linearized plots (anamorphoses) in the coordinates of the Freundlich equation display high coefficients of determination. The pronounced differences in distribution coefficients among rare earth ions with similar chemical properties—Kd (mL·g^−1^): Sm(III) 187 ± 19, Eu(III) 225 ± 18, Gd(III) 87 ± 9, Er(III) 1230 ± 98, Dy(III) 460 ± 37, Yb(III) 63 ± 11—highlight the selectivity of the material. This selectivity is apparently governed by variations in the stability of the surface complex compounds formed. Comparison of the IR spectra of material 5 before and after Ln(III) sorption reveals a decrease in the ν(C=O) frequency, confirming that extraction proceeds via a coordination mechanism involving formation of a covalent C–O–Ln bond. The relatively low functional group loading, as estimated from TGA data, further contributes to enhanced selectivity by limiting the number of available binding sites. Despite the similar chemical properties of Er(III) and Dy(III), the material demonstrates clear selectivity in their extraction under both static and dynamic conditions. Analysis of the dynamic breakthrough curves indicates the potential for separating Eu(III), Gd(III), Sm(III), and Yb(III) from Er(III) and Dy(III).

## 5. Patents

Patent RU 2842657. Konshina, D.N.; Spesivaia E.S.; Konshin V.V.; Lupanova, I.A. Method of producing silica with covalently immobilized 3-acyl-4-hydroxycoumarin. Effective date for property rights: 13 December 2024. Date of publication: 1 July 2025.

## Figures and Tables

**Table 1:** Sorption capacity and distribution coefficients of Gd(III), Dy(III), Er(III) (Eu(III), Sm(III), Yb(III).

Me(III)	A_max_, mmol·g^−1^, x ± ts/$\sqrt{n}$	Kd, mL·g^−1^, x ± ts/$\sqrt{n}$
Sm(III)	0.09 ± 0.02	187 ± 19
Eu(III)	0.12 ± 0.03	225 ± 18
Gd(III)	0.10 ± 0.02	87 ± 9
Er(III)	0.11 ± 0.02	1230 ± 98
Dy(III)	0.13 ± 0.03	460 ± 37
Yb(III)	0.10 ± 0.02	63 ± 11

**Table 2:** Dynamic distribution coefficient Gd(III), Dy(III), Er(III) Eu(III), Sm(III), Yb(III) in during individual preconcentration (*p* = 0.95; *n* = 3).

Kd, mL·g^−1^, x ± ts/$\sqrt{n}$					
Dy	Sm	Eu	Gd	Er	Yb
602 ± 65	392 ± 56	377 ± 48	317 ± 51	741 ± 51	180 ± 44

**Table 3:** Dynamic distribution coefficient Gd(III), Dy(III), Er(III) Eu(III), Sm(III), Yb(III) in during preconcentration from six-component systems (*p* = 0.95; *n* = 3).

Kd, mL·g^−1^, x ± ts/$\sqrt{n}$					
Dy	Sm	Eu	Gd	Er	Yb
560 ± 42	292 ± 41	247 ± 42	237 ± 38	707 ± 57	157 ± 34

## Data Availability

The original contributions presented in this study are included in the article and Supplementary Materials. Further inquiries can be directed to the corresponding author.

## Supplementary Materials

The following supporting information can be downloaded at: https://www.mdpi.com/article/10.3390/molecules31020369/s1, Figure S1: IR-spectrum 3-azidopropyl-silica; Figure S2: IR-spectrum 3-acyl-4-hydroxycoumarin-modified silica; Figure S3: IR-spectrum 3-acyl-4-hydroxycoumarin-modified silica + Er(III); Figure S4: IR-spectrum 3-acyl-4-hydroxycoumarin-modified silica + Dy(III).
