# Supplementary material for: New Strategy Based on Click Reaction for Preparation of 3-Acyl-4-hydroxycoumarin-Modified Silica as a Perspective Material for the Separation of Rare Earth Elements
Source: Molecules. 2026 Jan 20;31(2):369. doi: 10.3390/molecules31020369 (PMC12844081; doi:10.3390/molecules31020369)
Supplement: Supplementary file 1 [file molecules-31-00369-s001.zip › molecules-4059757-supplementary.pdf]

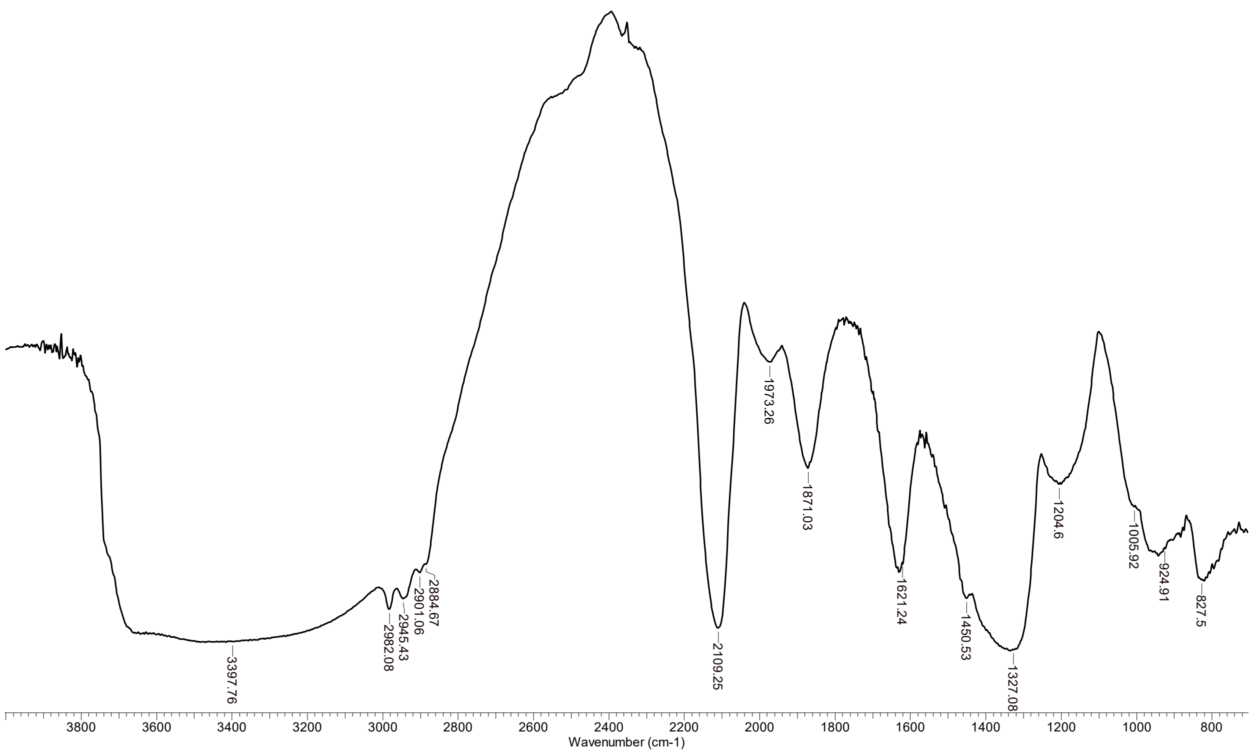

Fig.S1 IR-spectrum 3-azidopropyl-silica

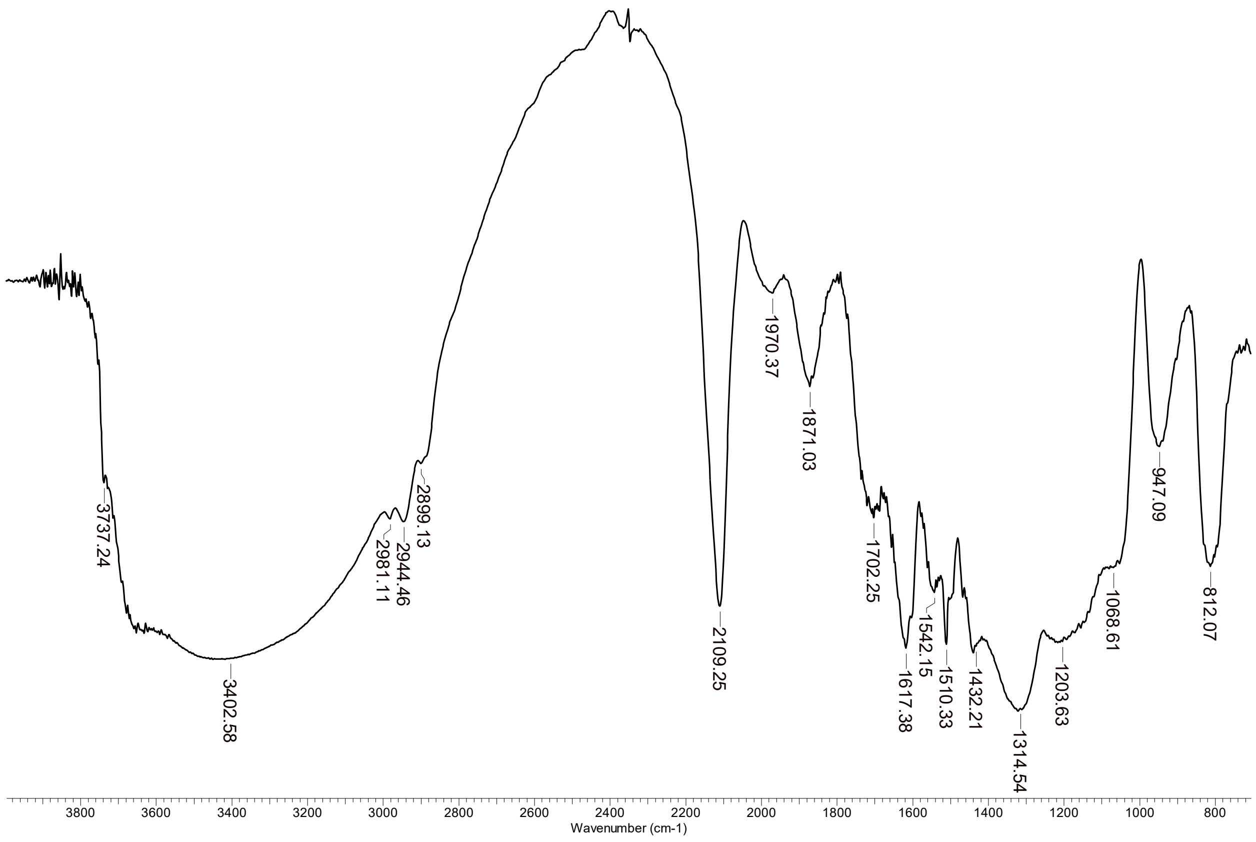

Fig.S2 IR-spectrum 3-acyl-4-hydroxycoumarin-modified silica

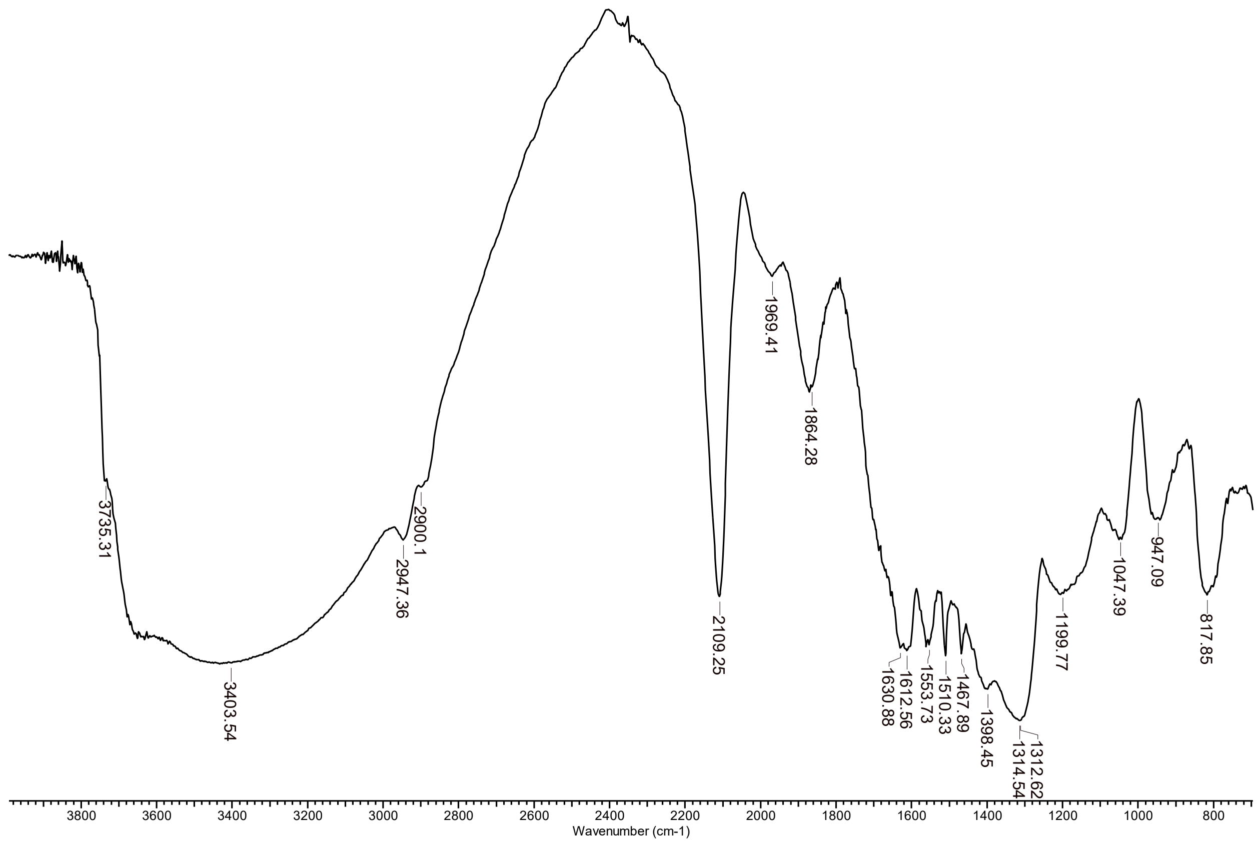

Fig.S3 IR-spectrum 3-acyl-4-hydroxycoumarin-modified silica + Er(III)

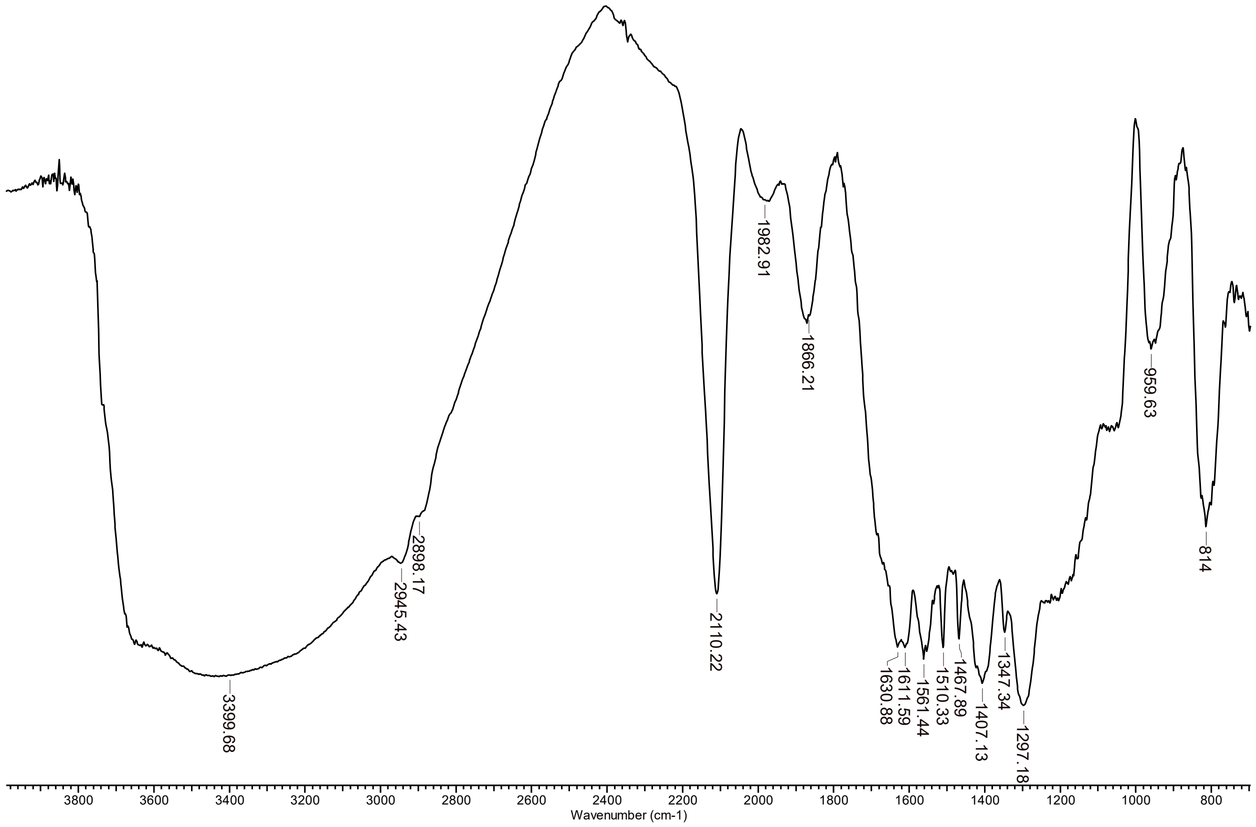

Fig.S4 IR-spectrum 3-acyl-4-hydroxycoumarin-modified silica + Dy(III)
